# Supplementary material for: The Influence of Acetate and Sodium Chloride Concentration on the Toxic Response of Electroactive Microorganisms
Source: Microorganisms. 2025 Sep 6;13(9):2077. doi: 10.3390/microorganisms13092077 (PMC12471985; doi:10.3390/microorganisms13092077)
Supplement: Supplementary file 1 [file microorganisms-13-02077-s001.zip › microorganisms-3774974-supplementary.pdf]

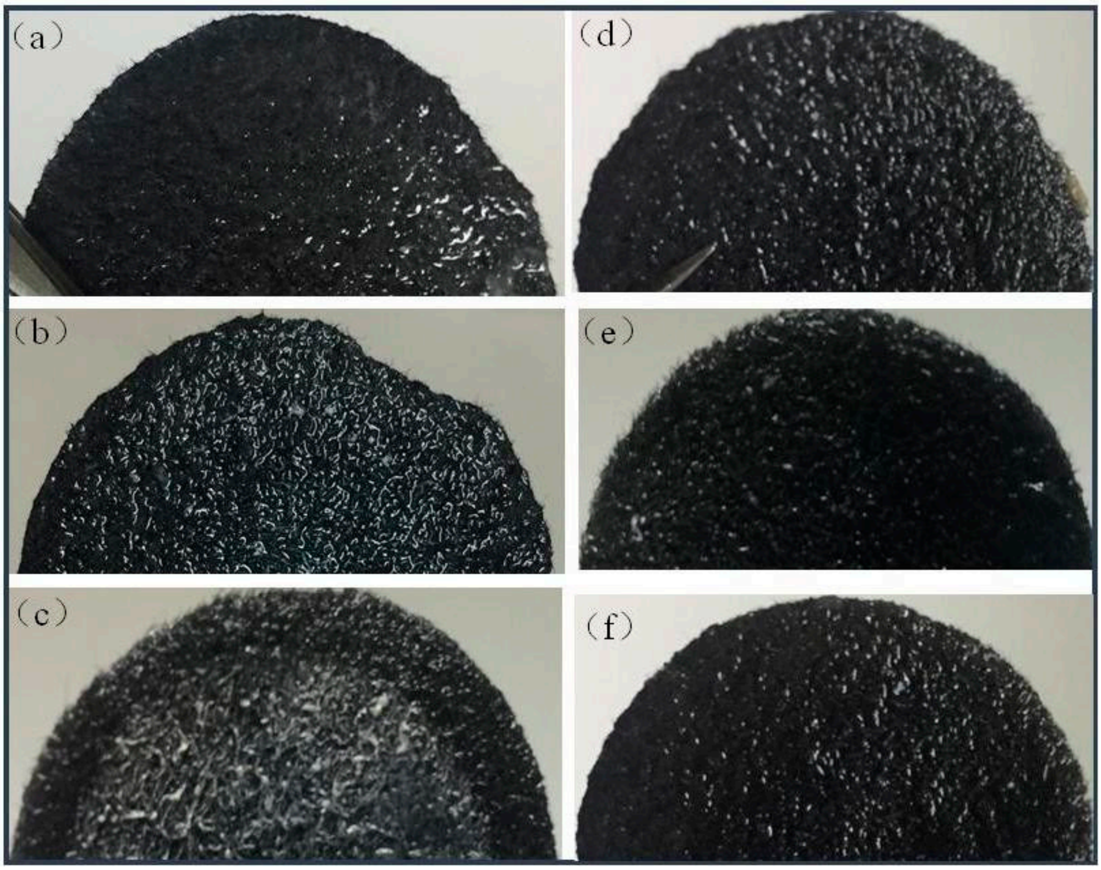

Figure S1. Images of anode under different acetate concentration of 0.5 g/L, 1 g/L, 5 g/L and sodium chloride concentration of 0.005 g/L, 0.0125 g/L, 5 g/L. (a) acetate concentration of 0.5 g/L; (b) acetate concentration of 1 g/L; (c) acetate concentration of 5 g/L; (d) sodium chloride concentration of 0.005 g/L; (e) sodium chloride concentration of 0.0125 g/L; (f) sodium chloride concentration of 5 g/L.

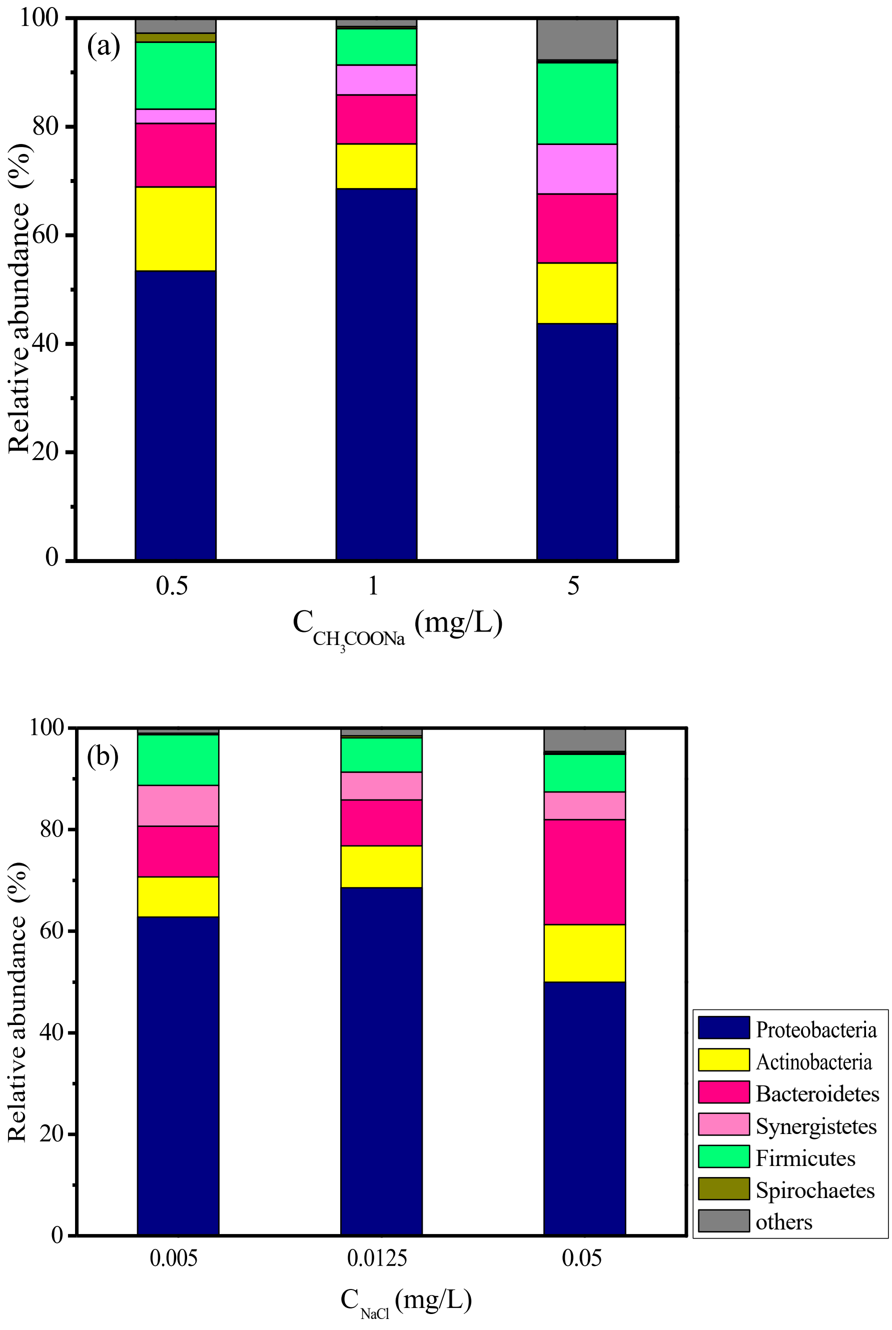

Figure S2. The content of anode bacteria at the level of phylum.
